# Phagocytosis of Microglia in the Central Nervous System Diseases

**DOI:** 10.1007/s12035-013-8620-6

**Published:** 2014-01-07

**Authors:** Ruying Fu, Qingyu Shen, Pengfei Xu, Jin Jun Luo, Yamei Tang

**Affiliations:** 1 Department of Neurology, Sun Yat-Sen Memorial Hospital, Sun Yat-Sen University, Number 107, Yan Jiang Xi Road, Guangzhou, 510120 Guangdong Province China; 2 Department of Neurology, Zengcheng People’s Hospital, Sun Yat-Sen University, Guangzhou, China; 3 Department of Neurology, School of Medicine, Temple University, Philadelphia, PA USA; 4 Key Laboratory of Malignant Tumor Gene Regulation and Target Therapy of Guangdong Higher Education Institutes, Sun Yat-Sen University, Guangzhou, China

**Keywords:** Microglia, Phagocytosis, Neurological diseases, Macrophages

## Abstract

Microglia, the resident macrophages of the central nervous system, rapidly activate in nearly all kinds of neurological diseases. These activated microglia become highly motile, secreting inflammatory cytokines, migrating to the lesion area, and phagocytosing cell debris or damaged neurons. During the past decades, the secretory property and chemotaxis of microglia have been well-studied, while relatively less attention has been paid to microglial phagocytosis. So far there is no obvious concordance with whether it is beneficial or detrimental in tissue repair. This review focuses on phagocytic phenotype of microglia in neurological diseases such as Alzheimer’s disease, multiple sclerosis, Parkinson’s disease, traumatic brain injury, ischemic and other brain diseases. Microglial morphological characteristics, involved receptors and signaling pathways, distribution variation along with time and space changes, and environmental factors that affecting phagocytic function in each disease are reviewed. Moreover, a comparison of contributions between macrophages from peripheral circulation and the resident microglia to these pathogenic processes will also be discussed.

## Introduction

Microglia, which constitute 10∼20 % of glia cells, are the resident macrophages of the brain and spinal cord and act as the main immune defense in the central nervous system (CNS). Since first described by del Rio-Hortega, microglia have attracted much attention in exploring their multifaceted meticulous functions, especially related to neuronal inflammation. Microglia are inactive under physiological conditions, with small cell body and highly ramified branching processes. In response to injury or pathogen invasion however, microglia transform into active phagocytic microglia [1], migrate, and accumulate at the site of injury through a process known as chemotaxis [2]. Being different to their resting phenotype, activated microglia are identified by their retracted processes and “amoeboid” morphology, release of both pro- and anti-inflammatory molecules, and high capacity for phagocytic removal of apoptotic cells and debris [3, 4]. Activated microglia are observed in nearly all kinds of neurological diseases, including neurodegenerative diseases such as Alzheimer’s disease (AD) [5], Parkinson’s disease (PD) [6], and amyotrophic lateral sclerosis (ALS) [7]; infectious and inflammatory diseases such as multiple sclerosis (MS) [8, 9]; stroke [10]; and traumatic [11] and radiation-induced brain injury [12].

In recent years, numerous papers have focused on microglial function of chemotaxis and secretion of pro- and anti-inflammatory cytokines, growth factors, chemokines, and neurotrophins; however, studies regarding microglial phagocytosis are rare. Recently, “phagoptosis” has been proposed as a form of cell death, caused by phagocytosis of viable cells [13]. It is also known as “primary phagocytosis.” It is provoked by exposure of “eat-me” signals and/or loss of “don’t-eat-me” signals by viable cells, causing their phagocytosis by phagocytes and resulting in self-destruction [13]. Phagocytosis is normally exerted by phagocytic cells, such as microglia and macrophages [13]. Whether microglial phagocytosis plays a beneficial or detrimental role in brain diseases remains controversial, though most researchers are in favor of the former claim since efficient clearance of tissue debris is critical in reconstruction and reorganization of neuronal networking after an injury in the brain [14–16]. The crucial beneficial role of microglial phagocytosis in axon regeneration and in restoration of the microenvironment has also been shown during the recovery of an acute brain injury.

In this article, we will review the characteristics of phagocytic microglia in some brain diseases and discuss quantity variation along with time and localization, receptors and following signaling pathways that mediated microglial phagocytosis, and correlative influential factors. In addition, the relative contribution made by resident microglia and hematogenous macrophages during disease processes is also reviewed.

## Receptors Involved in Microglial Phagocytosis

**Fig. 1:**
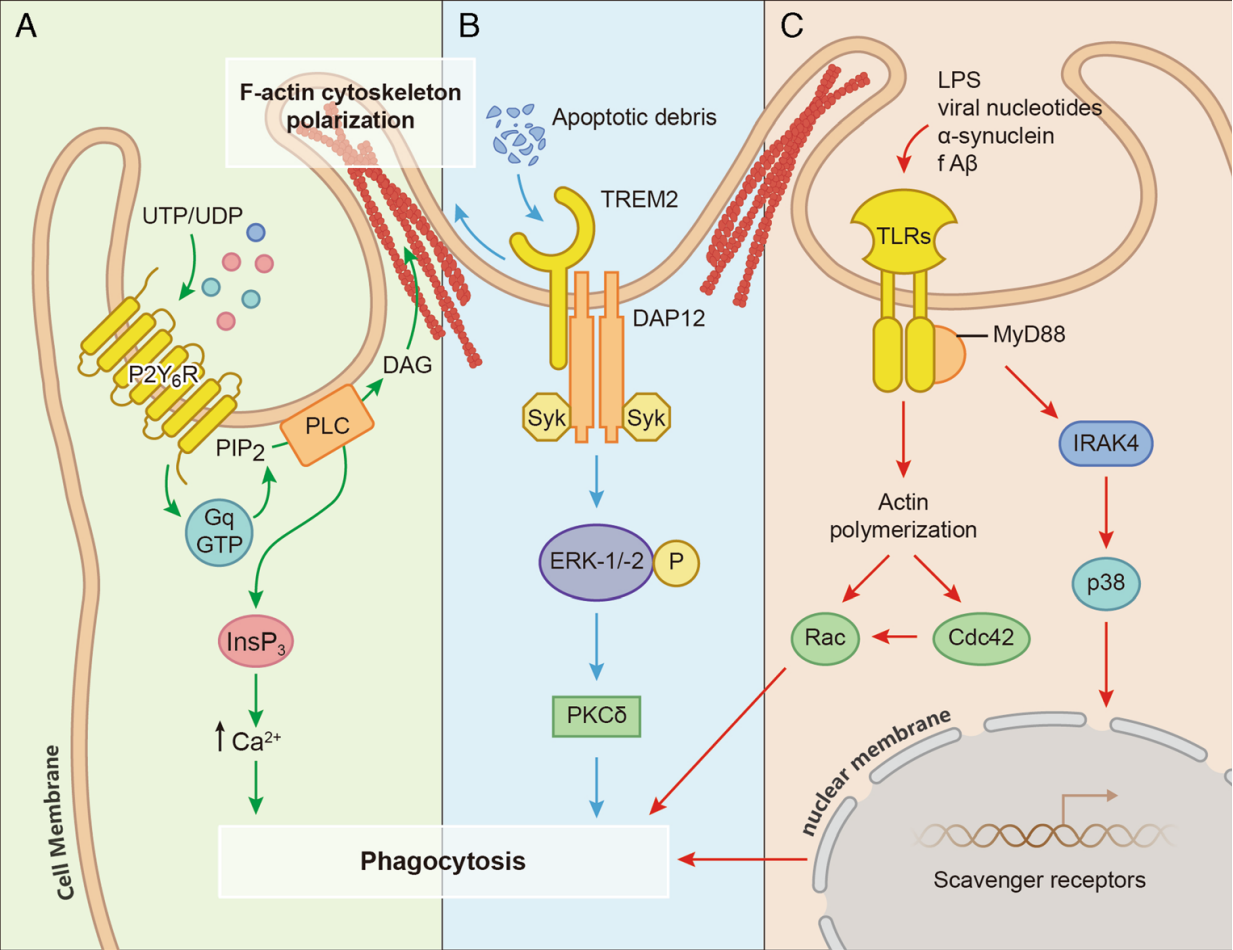
Signaling pathways involved in microglial phagocytosis. *A* Extracellular nucleotides, such as UDP and UTP, trigger microglial phagocytosis through P2Y6R/PLC/InsP3 pathway. *B* Apoptotic debris induces phagocytosis via TREM-2/DAP12/ERK/PKC pathway. *C* Endogenous or ectogenic detriments, such as LPS, viral nucleotides, α-synuclein, and f-Aβ, provoke phagocytosis by microglia via TLRs through activation of MyD88-dependent IRAK4/p38/scavenger receptors pathway or MyD88-independent actin-Cdc42/Rac signaling pathway

The activity of microglial phagocytosis relies on specific receptors expressed on the cell surface and downstream signaling pathways that contribute to the reorganization of actin protein and engulfment of harmful microparticles (Fig. 1; Table 1). Microglial phagocytosis may need different types of receptors to initiate function [17]. In general, there are two distinctive types of receptors, one with a high affinity to bind to foreign microbial pathogens, such as Toll-like receptors (TLRs), and another recognizing apoptotic cellular substances, such as triggering receptor expressed on myeloid cells 2 (TREM-2). Besides these two types, some receptors including Fc receptors, complement receptors [18], scavenger receptors (SR), pyrimidinergic receptor P2Y, G-protein coupled, 6 (P2RY6), macrophage antigen complex 2 (MAC-2), mannose receptor [19], and low-density lipoprotein receptor-related protein (LRP) receptor also participate in microglial clearance of misfolded, apoptotic cells and dead neurons in both acute and chronic brain injury [20].

**Table 1:** Receptors involved in microglial phagocytosis

Receptors	Stimulus	Cell types expressed	Diseases/animal models	References
TLRs	α-Synuclein	Microglia	alpha-Synucleinopathies, Alzheimer’s disease	[23, 120–122]
	Fibrillar Aβ	Astrocytes		
	Bacterial strains	Oligodendrocytes	CNS infections, spinal cord injury, and neuronal injury	
	LPS	Neurons		
TREM-2	Amyloid protein	Microglia	Alzheimer’s disease	[34, 123]
		Macrophages	Nasu-Hakola disease	
		Monocyte-derived dendritic cells	Multiple sclerosis/EAE	
		Osteoclasts		
Fc receptors (FcγR)	α-Synuclein	Neurons	Parkinson’s disease	[124, 125]
		Astrocytes	Multiple sclerosis	
		Microglia	Alzheimer’s disease	
		Oligodendrocytes		
Complement receptors (CR3 (CD11b/CD18), CR4 (CD11c/CD18))	Degenerated myelin	Microglia	Alzheimer’s disease	[126, 127]
	Aβ	Macrophages	Wallerian degeneration	
	Apoptotic cells		Multiple sclerosis	
Scavenger receptors (SR-A, SR-BI, CD36, RAGE)	Degenerated myelin	Microglia, astrocytes, mato cells, cerebral microvascular endothelial cells, cerebral arterial smooth muscle cells, retinal pigment epithelial cells	Multiple sclerosis/EAE	[128–131]
	fAβ, thrombospondin-1		Amyotrophic lateral sclerosis	
	Anionic polysaccharide		Alzheimer’s disease	
	Polynucleotides			
	Chemically modified proteins			
	Apoptotic cells			
	Bacteria			
P2RY6	ATP and other nucleotides	Microglia	Neuropathic pain	[37, 132]
Galectin-3/MAC-2	Degenerated myelin	Microglia	Traumatic brain injury	[133, 134]
		Macrophages	Spinal cord injury	
		Schwann cells	Multiple sclerosis/EAE	
LRP receptors (low-density lipoprotein receptor-related protein-1)	LPS or Aβ	Neurons	Alzheimer’s disease	[135–137]
		Brain capillary endothelium		
Phosphatidylserine receptors (phosphatidyl-serine-specific receptor)	Apoptotic and necrotic neurons	Macrophages	Alzheimer’s disease	[138, 139]
		Fibroblasts		
		Epithelial cells		
		Dendritic cells		
Mannose receptors	Soluble immune components, cell debris, cytotoxic substances, myelin glycoproteins, degrading enzymes, microbial ligands	Astrocytes	Ischemia	[140, 141]
		Perivascular		
		Meningeal		
		Choroid plexus macrophages		

### TLRs

TLRs are a class of proteins that play a key role in the innate immune system and the digestive system. TLRs are single, membrane-spanning, non-catalytic receptors usually expressed in sentinel cells, such as macrophages and dendritic cells, that recognize structurally conserved molecules derived from microbes. Once these microbes have breached physical barriers such as the skin or intestinal tract mucosa, they are recognized by TLRs, which activate immune cell responses. TLR1–9, which belong to interleukin (IL)-1R super-family, expressed exclusively on antigen presenting cells including microglia [21], macrophages, antigen presenting dendritic cells, and cerebral parenchyma cells which contain neurons, oligodendrites, and astrocytes. TLRs not only trigger the recognition of pathogen-associated molecular patterns, such as LPS or viral nucleotides, but also recognize danger-associated molecular patterns, such as deposited amyloid β (Aβ) fibril and α-synuclein [22, 23]. TLRs are also implicated in a variety of cerebral disorders, including bacterial or viral infections; neurodegenerative disorders such as AD; inflammatory demyelinating disorders such as MS; spinal cord injury (SCI); and in development or physiological processes such as neurogenesis, learning, and memory [22–25]. TLRs and TLR-dependent signaling pathways are involved in antibacterial immunity and restricting viral infection in CNS infection. Of note, TLR2 and TLR4 mediate brain injury and subsequent inflammation after ischemic stroke [25–28]. TLR4-, TLR2-, and TLR9-dependent signaling pathways are involved in mediating microglial phagocytosis of neurotoxic Aβ deposit in AD brain and exert a protective role in nerve regeneration [28–30]. It has been reported that TLRs regulate phagocytosis through myeloid differentiation factor 88(MyD88)-dependent and MyD88-independent signaling pathways. The MyD88-dependent pathway is triggered by TLRs through activation of IL-1 receptor-associated kinase (IRAK)-4 and p38, resulting in up-regulation of scavenger receptors [31]. On the other hand, TLRs also regulate phagocytosis by MyD88-independent actin-Cdc42/Rac pathway [32].

### TREM-2

TREM-2 is a kind of pattern receptor specific for polyanionic and locates mainly on the cell surface of osteoclasts in bones and in microglia of the CNS [8, 33]. In addition to up-regulating the synthesis of chemokines and mediating protective phagocytosis of apoptotic cell debris, activation of TREM-2 receptors suppresses secretion of pro-inflammatory factors such as cytokines and ROS [8, 33]. Clinical observation showed that administration of specific agonist or antibody of TREM during the effector phase of MS led to a more severe immune response and resulted in more extensive demyelination [34]. TREM-2 on microglia via binding with DNAX-activation protein 12 (DAP12), an ITAM-containing adaptor protein, triggers the reorganization of F-actin and phosphorylation of ERK/MAPK, mediating the clearance of apoptotic neurons [34, 35]. Nasu–Hakola disease, a systemic bone cystic disorder with progressive presenile dementia followed by extensive sclerosis in the front-temporal lobe and the basal ganglia, occurs due to genetic mutation of TREM-2 and DAP12 resulting in aberrant TREM-2/DAP12 signaling pathway [36].

### P2Y6

P2Y6 receptor, a member of the G-protein-coupled receptor family, is actively responsive to UDP and partially responsive to UTP and ADP. The study of P2Y6 receptor has gained increasing attention during the past several years since the elegant demonstration that P2Y6 receptor triggers the UDP-evoked microglial phagocytosis [37]. In other words, UDP, which is released from injured neurons after trauma or ischemia, acts as “eat me” signal and meditates the P2Y6-dependent phagocytosis. P2Y6, when combined with UDP, activates phospholipase C (PLC) which in turn causes the synthesis of inositol 1,4,5-trisphophate (InsP3) and triggers the booted release of Ca^2+^ from InsP3-receptor-sensitive stores [37]. In addition to triggering the intracellular Ca^2+^ over-loading, P2Y6-receptor-dependent signaling pathway also triggers actin cytoskeleton polarization to shape filopodia-like protrusions, thus facilitates the engulfment of cell debris [37].

## Microglial Phagocytosis in CNS Diseases

### Microglial Phagocytosis in AD

AD is a progressive neurodegenerative disease characterized by progressive memory loss, change in personality, and dementia. The distinctive pathological hallmarks of AD are the presence of extracellular plaques of Aβ peptide and intracellular neurofibrillar tangles of tau protein. Activated microglia, reactive astrocytes, and profound neuronal loss are also evident [38]. Human studies disclosed that the accumulation of Aβ deposition in the brain correlated well with cognitive impairment and neuronal loss [39]. Microglial cells play a crucial role in removing Aβ in different ways. Using 3D reconstruction of microglia and amyloid in an animal model of AD, Stalder et al. demonstrated that clusters of activated microglia contained lysosomes or vacuoles in close vicinity of the dense core plaques [40]. An in vitro study of pulse-chase experiments with Cy3-labeledα_2_-macroglobulin (α_2_M) and Cy3-labeled Aβ microaggregates in the cultured microglia showed that rapid uptake of both α_2_M and Aβ microaggregates took place within 15 min, and those vacuoles could reach the acidic endosomes and lysosomes within 1 h. After 4 h, most internalized α_2_-M were degraded or released from cells. Interestingly, microglial cells became engorged with undigested Aβ after continuous incubation with Aβ aggregation for 4 days, suggesting a slow rate of degradation of the fibrils [41]. Bamberger et al. reported the identification of the multicomponent receptor complex of fibrillar Aβ (fAβ) on microglial cell surface [42]. The principle constituents of the complex are CD36, CD47 (also termed integrin-associated protein), and the α6β1-integrin [43]. This receptor complex mediates the adhesion of Aβ fibrils (fAβ) to microglia and elicits phagocytosis. The bond of fAβ to the ensemble of receptors leads to activation of tyrosine kinase-mediated signal transduction cascades, which then results in a respiratory burst and production of IL-1β [42]. Koenigsknecht and Landreth provided data arguing that the cell surface receptor complex-mediated engagement of fAβ was driven principally by a novel β_1_-integrin-dependent mechanism, but not by classical phagocytic mechanisms mediated either by Ig receptors or the complement receptor 3 [43]. Mo/Hu APPswe PS1dE9 mice are capable of producing numerous Aβ deposits in the brain. C3H/HeJ mice are mutated with destructive *TLR4* gene (*Tlr* ^*Lps*-*d*^), which causes inactivation of TLR4 by LPS. Mo/Hu APPswe PS1dE9 mice with TlrLps-d/TlrLps-d genotype were developed from Mo/Hu APPswe PS1dE9 mice mating with C3H/HeJ mice. These animal models exhibited an increase of cerebral Aβ load, which suggested that TLRs, particularly TLR-2 and TLR-4, were involved in the phagocytosis of Aβ deposit in brain parenchyma and thus exerted a protective role in AD patients [29]. FcR-mediated phagocytosis and complement activation also play a critical role in removal of plaques from the AD brain [30]. Additionally, monocyte chemotactic protein-1 (MCP-1/CCL2), coupled with its binding receptor, CC-chemokine receptor 2, was crucial in neuroinflammatory response that affected disease process in a mouse model of AD [44–46]. CCL2-deficient AD mice (APP/PS1 mice) showed decreased microglial phagocytosis for both monomeric and oligomeric Aβ42 and accelerated Aβ deposits and oligomers [46]. Interestingly, transgenic overexpression of CCL-2 in APP/CCL2 mice dramatically facilitated Aβ uptake and subsequent intracellular Aβ oligomerization and resulted in progression of neurocognitive decline [45]. The exact role of CCL-2 in AD remains further study. Koenigsknecht-Talboo and Landreth have studied that the pro-inflammatory environment of the AD brain impaired the microglial capacity of removal of Aβ, whereas anti-inflammatory factors enhanced Aβ clearance [47]. Microglia treated with pro-inflammatory cytokines such as LPS, IL-1β, TNF-α, IFN-γ, MCP-1, and CD40L suppressed fAβ-stimulated microglial phagocytic activity in vitro [47]. This effect was even significant during activation of the CR3 or fAβ receptor complex, but did not affect IgG- or FcR-mediated phagocytosis [47]. On the other hand, when BV2 cells were incubated with anti-inflammatory cytokines in a pro-inflammatory milieu, these phagocytes exhibited a stronger competence to uptake fibrillar Aβ [47]. Anti-inflammatory cytokines, such as IL-4, IL-10, cyclooxygenase (COX) inhibitors, ibuprofen, or an E prostanoid receptor antagonist, blocked NFκB-dependent stimulation of COX-2 expression and prostaglandin E2 (PGE2) production or its cascade pathways to eliminate the inhibitory activity of pro-inflammatory cytokines and consequently restored fAβ-stimulated phagocytic response [42, 43, 47, 48]. These observations are consistent with previous demonstration that deletion of PGE2 EP2 receptor in animal model of familial AD resulted in a marked reduction of Aβ plaque burden and enhanced phagocytosis [49].

Although microglia exhibit “activated” phenotype, they are presumably unable to efficiently uptake Aβ plaque to prevent the neurodegenerative progression of AD patients [42]. Treatment with antibodies against Aβ peptide to PDAPP transgenic mouse model of AD dramatically reduced Aβ deposit by facilitating clearance of preexisting amyloid rather than by simply preventing new plaques formation [50]. Recent studies showed that treatment with anti-Aβ antibodies may be effective in reducing plaque burden and reversing memory deficits in mouse model for AD [51, 52]. Therefore, modulating phagocytosis and degradation of fAβ by microglia might be a potential treatment of AD.

### Microglial Phagocytosis in Trauma

Traumatic brain injury (TBI) and SCI are the most common forms of CNS traumatic injury. Much attention has been given to their pathological mechanisms. SCI occurs from a primary mechanical insult on the spinal cord, resulting in damage of neurons and axons [53], followed by a second-wave injury. Synthesis of free radicals and nitric oxide, as well as glutamate receptor-associated excitotoxicity and release of protease, was observed in the process of secondary injury. Microglia, together with infiltrated peripheral macrophages and T cells, have long been proposed to be involved in the delayed secondary injury. Microglia, as the principal immune effectors in the brain, undergo a conversion into a reactive pro-inflammatory phenotype in neurotrauma rapidly [54]. ED1-positive microglia began to be detected at 6 h after SCI and mostly located close to the lesion epicenter [55]. Within 48 h post-injury, the ED-1 positive microglia showed the tendency of a higher density and a broader macrophages/microglia infiltrating extension [55]. It is striking that a higher density of macrophages/microglia in the gray matter than white matter continued to be observed [55]. Since activated microglia and infiltrated peripheral macrophages exhibit the similar morphology, gene expression, as well as surface and endocellular markers, there is still no discriminating cellular markers to distinguish these two cell types in the CNS so far.

Accumulating evidence has shown that microglia play a pivotal role in the process of spinal cord regeneration and degeneration during the so-called secondary injury [11]. Microglia, when exposed to myelin in vitro, rapidly transformed from ramified to activated amoeboid morphology. These activated microglia synthesized and released a wide range of pro-inflammatory cytokines, chemokines, anti-inflammatory cytokines, neurotrophic factors, and phagocytosed the cellular debris.

In a dog model for SCI, a significant myelinophagia occurred 5 days after trauma, which was concomitant to the phagocytic activation of microglia [56]. An enhanced phagocytic capacity of single microglial cell was observed, particularly in the early 5 days, but the number of phagocytes did not change [56]. In this study, the capacity of microglial phagocytosis was measured by flow cytometric assay using FITC-labeled *Staphylococcus aureus*. The bacteria were added into microglial cell suspension to determine the percentage of microglia with phagocytic phenotype. The enhanced phagocytic property was proposed to be required to remove the injured neurons, axons, and myelin sheaths. However, in the later stage, number of microglia with phagocytic phenotype increased, accompanied by less activity of phagocytosis of individual cell [56]. The rapid clearance of degenerated myelin is beneficial to trigger efficient remyelination.

Following neuronal axon injury, a classical response in the CNS is to remove or “strip off” synapses from the affected cell body, which is mediated by glia cells. The early studies by Cullheim and Thams elegantly demonstrated that major histocompatibility complex (MHC) class I molecules and MHC class I receptors played a key role in synaptic plasticity and nerve-regeneration after axonomy [57]. Synaptic stripping preferred to remove excitotoxic glutamatergic rather than the inhibitory glycinergic and GABAergic nerve terminals, suggesting a beneficial effect for the repair. Functional MHC class I took part in normal “stripping” by specifically keeping the inhibitory influence on injured motoneurons. β2-Microglobulin, a subunit of MHC class I and transporter associated with antigen processing 1 knockdown or knockout mice, displayed remarkable elimination of synapses, resulting in less synaptic terminals remaining on the surface of affected neurons [57]. This observation was supported by a recent study by Sabha and colleagues showing a robust up-regulation of MHC I class after axonomy in different mice strains [58]. C57BL/6J mice with a low level of MHC I expression showed poor axon regeneration and slow synaptic stripping from motor neuron surface following peripheral nerve axonomy [58]. Additionally, A/J mice after axonomy exhibited significant elevation of MHC I expression and stronger regenerative potential, accompanied with more intense of synaptic elimination [58]. Zanon and colleagues indicated that IFN-β treatment notably increased MHC class I expression after sciatic nerve crush in mice [59].

A recent study by Gitik et al. demonstrated that CD47 (also known as integrin-associated protein), expressed on myelin, when reacting to immune inhibitory receptor signal regulatory protein-α (SIRP-α) on microglia, notably down-regulated myelin phagocytosis by microglia in vitro [60]. This finding suggested that CD47 on one hand exerts a neuroprotective function by protecting normal intact myelin from activated phagocytes, while on the other hand impedes normal phagocytosis therefore is unfavorable to the repair of lesioned neurons. Previous experiments have demonstrated that complement receptor-3 (CR3/MAC-1) signaling pathway was involved in the clearance of degenerated myelin [61]. CR3/MAC-1, as a member of integrin superfamily, is composed by α_M_ and β_2_ subunit [62] and mediates myelin phagocytosis via binding to both complement (iC3b) and noncomplement ligand (e.g., fibrinogen and I-CAM) [61]. A study by Rotshenker and colleagues demonstrated that CR3/MAC-1 activated guanine nucleotide exchange factors that facilitated the conversion of inactive K-Ras-GDP to active K-Ras-GTP, which then led to activation of PI3K and myelin phagocytosis [63]. Treating microglia with TNF-α suppressed myelin phagocytosis mediated by CR3/MAC-1 [64]. Scavenger receptor AI/II (SRAI/II) also participates in myelin clearance after trauma. However, the relative potential contribution of CR3/MAC-1 to myelin phagocytosis is two- or three-folds more than that of SRAI/II [65]. Microglia immunophenotypical characterization in dogs with SCI revealed a remarkable upregulation of mediators for phagocytosis, including ICAM-I, CD14, CD44, and CD45, and the increasing of expression intensity of these surface molecules significantly enhanced the removal of tissue debris [56].

Myelin structures, such as Nogo-A, myelin-associated glycoprotein, oligodendrocytes myelin glycoprotein, the transmembrane semaphoring 4D (Sema4D/CD100), and ephrinB3, translocate to cell surface on injured myelin and act as inhibitors of axonal regeneration [66]. Therefore, efficient microglial phagocytosis of damaged myelin and cell debris is beneficial for the survival of injured neurons and myelin regeneration in acute SCI and TBI [56, 67]. The enhanced microglial phagocytosis of myelin debris was demonstrated dramatically to decrease dead cells and myelin debris and result in prominent functional recovery [61].

### Microglial Phagocytosis in Ischemic/Stroke

Stroke is the most common disease in the CNS among the elderly. Rapid infiltration of polymorphonuclear neutrophils (PMNs) and peripheral monocytes/macrophages and abundant activation of resident microglia are observed in the injured brain post-ischemia lesion [68, 69]. In an in vivo study, Schilling et al. demonstrated that both resident microglia and hematogenous macrophages made contributions to debris clearance post-cerebral infarction, while microglia played a more major role than did macrophages [70]. Activated microglia were observed to rapidly migrated into the infarction area and elicited phagocytic response at day 1 after ischemia [71, 72], but no peripheral infiltration was seen [70]. The total number of activated microglia showed an increased tendency in the following several days and reached maximum at day 10 after transient focal cerebral ischemia [70], whereas blood-borne macrophages began to occur in the damaged lesion at day 4, increased to the peak number in the following 3 days, and began to decrease until 2 weeks after ischemic insult [70]. Strikingly, phagocytic microglia only accounted for one quarter of the total activated microglia. This phenotype of microglia rapidly had their maximum number as early as day 1 and stayed at the same level within the following days until the hematogenous macrophages infiltrated [70]. These findings lead to the hypothesis that participation of microglia in the debris clearance takes place in the early stage while the assistance from blood-borne macrophages infiltration in the infarction zone occurs later.

In vivo study showed that PMN rapidly infiltrated and accumulated in ischemic-injured brain as early as day 1 after cerebral ischemia [69, 73]. Application of PMNs onto post-oxygen–glucose deprivation organotypic hippocampal slice cultures (OGD-OHCs) led to a remarkable exacerbation of neuronal damage, characterized by severe loss of axons and dendrites, as well as the appearance of apoptotic or necrotic neurons containing subcellular material at day 1 [69]. Interestingly, the co-application of PMNs and microglia onto the OGD-OHCs significantly reduced the PMN-induced damage to neurons, which was presumably due to direct engulfment of those viable, motile, and nonapoptotic PMNs by microglia [69]. Uptake of PMNs by microglia significantly reduced the release of pro-inflammatory mediators, such as cytokines and ROS, and was associated with the upregulation of TGF-β in microglia, both of which exerted a beneficial role in the injured brain [69]. The exact mechanisms and signals involved in recognition and uptake of PMN by microglia are not yet defined. The capacity of microglia to engulf PMN can be inhibited by TNF-α. Furthermore, microglia also elicited phagocytic response through interaction with cell surface receptors, for example, osteopontin (OPN), an adhesive glycoprotein [74]. It has previously been accepted that OPN acts only as a chemoattractant after ischemic insult, but recently, it has been reported that OPN also takes part in the phagocytosis of the cell debris [75]. Elevated expression of OPN protein was seen mainly along the membranes lining after ischemic stroke; thus, researchers concluded that OPN elicited phagocytic response of fragmented debris selectively [74].

Both microglia and hematogenous macrophages contribute to clearance of cell debris generated in the context of ischemia. The efficient phagocytosis of debris is beneficial for axon regeneration. In addition, phagocytosis by microglia/macrophages exerts favorable effect through engulfment of infiltrated PMN. Depletion of PMN subsequently converts the microenvironment from pro-inflammatory to anti-inflammatory, which reduce neuronal injury in the process of disease. In general, microglial phagocytosis might exert beneficial effect in ischemic brain.

### Microglial Phagocytosis in PD

PD is characterized by progressive dopaminergic neuronal loss in substantia nigra and nerve terminals in the striatum, resulted in bradykinesia, static tremor, instability of gait and posture, and muscle stiffness. Overexpression of extracellular α-synuclein is a well-known etiological pathology of PD [76]. Microglia treated with monomeric α-synuclein exhibited an enhanced phagocytic activity in vitro, in a both time- and dose-dependent manner [77]. In this study, microglial phagocytosis was evaluated by the activity of microglia in ingesting extracellular fluorescent microspheres. The exact mechanism underlying in monomeric α-synuclein induced phagocytosis by microglia is still an open question. Studies have confirmed the involvement of CR3, α6β1, and CD47, which are important components of receptor complex in clearance of Aβ [77]. In comparison, aggregated α-synuclein inhibited phagocytosis of cell debris and dead neurons not only by antagonizing monomeric-facilitated clearance but also through decreasing the basal microglial phagocytic capability [77]. It is also reported that microglia were capable of phagocytosing and degenerating neuromelanin (NM) released from degenerated dopaminergic neurons. Microglia co-cultured with NM revealed a rapid transform from a ramified to an amoeboid morphology and were engaged in attaching to and engulfing NM particles [78]. In a 6-hydroxydopamine (6-OHDA) model of PD, phagocytic microglia (CD68 positive) were found to surround the intact tyrosine hydroxylase-positive dopaminergic substantia nigra pars compacta (*SNc*) neurons to remove damaged NM particles in the *SNc* ipsilateral to the lesion side at day 3. At day 7, an increasing number of microglia were confined to the medial area of SNc to contact with intact dopaminergic neurons, as well as apoptotic cells. The number of microglia increased in a time-dependent manner and reached significance at day 9 and had their maximum at day 15 post-6-OHDA lesioning. Microglia were found to localize in the medial and lateral part of ipsilateral SNc. A vast proportion of phagocytic microglia (CD68 positive) were seen to adhere to and engulf degenerated dopaminergic neurons and axons [79]. Using proteomic technology, Liu et al. have shown that a variety of types of membrane proteins were potentially involved in the internalization of α-synuclein [80]. Clathrin was demonstrated to play a critical role in the endocytosis of aggregated α-synuclein, probably in a receptor-ligand sequestration-related manner [80], but the exact mechanism needs further study. Recently, TLR4 signaling pathway is demonstrated to mediate α-synuclein phagocytosis and exert a beneficial role in deferring disease progression both in vivo and in vitro [81]. In in vivo study of transgenic murine model of α-synucleinopathies (ASP), mice overexpressed human α-synuclein (hAS) with TLR4 deficiency (AS/TLR4^−/−^) exhibited severer neuronal loss, motor disability, and predominant reduced phagocytic activity than those with normal TLR4 expression(AS/TLR4^+/+^). Counterstaining of anti-hAS and CD11b showed abundant hAS-positive structure appeared in the cytoplasm of CD11b-positive microglia in AS/TLR4^+/+^ mice; on the contrary, hAS-positive structure was found only outside microglia in AS/TLR4^−/−^ mice. By further study with immunogold labeling for AS in the brain of transgenic mice, microglia in AS/TLR4^+/+^ mice showed abundant gold particles in phagocytic cytoplasmic organelles, while fewer gold particles were found in microglia in AS/TLR4^−/−^ mice [81]. Numerous studies also displayed that C1q-mediated pathway [82] scavenger receptors [83] and MAC-1 [78] are also involved in microglial endocytosis of α-synuclein. Over-expression of human wild type and mutant a-synuclein(A30P and A30T) in BV2 cells resulted in downregulation of phagocytosing bioparticles and a marked low lysosomal associated protein 1 expression, accompanied with elevated COX-2 and proinflammatory cytokines such as PGE2 [84].

The rate of internalization and subsequent intracellular degradation of extracellular α-synuclein aggregates were compared in the major brain cell types of neurons, microglia, and astrocytes. The finding demonstrated that all these three types of cells were capable of clearing α-synuclein. Among them, microglia showed to be the most effective [85]. Whether microglial phagocytosis of α-synuclein favors or harms the process of PD is still under debate. Zhang et al. argued that internalization of α-synuclein took a central role in dopaminergic neurotoxicity through activation of NADPH oxidase and subsequently oxidative stress [86]. However, as mentioned above, impaired microglial phagocytic capacity by ablation of TLR4 in ASP mouse model led to aggregation of extracellular α-synuclein and accelerated neurodegeneration. In brief, the role of microglial phagocytosis on PD remains further investigation.

### Microglial Phagocytosis in ALS

ALS is the most common progressive neurodegenerative disorder that selectively affects motoneurons in the CNS. Its remarkable characteristic is the simultaneous death of lower and upper motor neuron, leading to progressive muscle weakness and atrophy. Patients suffering from ALS usually end up with death from respiratory paralysis within 2 to 5 years of onset. Although ALS is overwhelmingly a sporadic disorder, genetic studies have established that mutations in the Cu/Zn superoxide dismutase 1 (SOD1) gene are the most well-known cause of familial ALS [87, 88]. Studies have shown that microglia has an important function in propagation of the disease process both in sporadic and familial ALS [89, 90] and in the transgenic animals overexpressing human mutant SOD1 (hmSOD1) [91]. By analysis of autopsy cases of ALS, increased numbers of macrophages were observed in the regions with motor neuron loss, such as lower motor neuron XII, upper motor neuron beta cells, spinocerebellar inferior olivary nuclei and red nuclei, somatosensory caudate nuclei and thalamus, cerebral cortex amygdaloid, and the ventral horn of the spinal cord [90]. This observation is supported by a recent experimental study which argued that activated microglia aggregated in the anterior horn of the lumbar spinal cord, particularly around impaired motor neurons [92]. This study also demonstrated that activated microglia attached to somata of motoneurons and exhibited phagocytic properties as early as presymptomatic stage [92]. Interestingly, activated microglia were visualized not only in regions where there was severe motor neuron loss but also in areas of mild motor neuronal damage [90]. However, the exact role of microglia in motor neuron degeneration remains unclear. In transgenic hmSOD1 (G93A) rat, it has been shown that CD11b-positive macrophages/microglia accumulated both in the ventral horn and more distally at the peripheral nerve during pre-symptom stage. As the disease progressed, microglia aggregated in the ventral horn and became more prevalent. These focal aggregates of microglia were observed often near but never fully encapsulating NeuN-labeled neurons. However, after onset of the symptoms, microglia labeled with CD68 and MHC class II (markers of activated microglia with phagocytic activity) began to be observed in the ventral horn. Therefore, microglia may cause damage to nearby motor neurons through secretion of proinflammatory factors at the early stage of ALS, while play a neuroprotective role through phagocytosing the degenerated debris following clinical onset [91].

TLR-dependent signaling pathways that modulate microglial phagocytosis play a key role in Wallerian degeneration (WD) and ALS. Injection of TLR-2 and TLR-4 enhanced myelin debris clearance and repairment of locomotor function after sciatic nerve lesion [93]. Blockade of TLRs and downstream signaling MyD88 decreased axons debris removal and delayed neuron regeneration [93]. MCP-1, macrophage inflammatory protein-1α, and interleukin-1β were reported to be involved in the progress of WD. Injection of these proinflammatory cytokines into the sciatic nerve led to rapid myelin damage and elevated macrophage/microglial phagocytic activity in WD [94]. Efficient removal of degenerated neurons or cell debris is necessary to rebuild beneficial environment for neuronal regeneration in degenerated disease. In response to neuronal cell death, microglia become activated and aggregate around the lesioned area, exhibiting potent phagocytic activity [95]. Enhancement of myelin phagocytosis by microglial/macrophages favors neuron regeneration and restoration of locomotor function.

### Microglial Phagocytosis in MS/EAE

MS is a chronic neurological disorder of the CNS which leads to nontraumatic disability among young adults. The pathological hallmarks of MS are extensive demyelination and formation of sclerosis plaques in cerebral cortex and spinal cord, which contribute to irreversible neurological injury. Experimental allergic encephalomyelitis (EAE) and Theiler’s murine encephalomyelitis virus infection are the two most commonly used animal models of human MS. Immune effector cells including reactive microglia and hematogenous macrophages, as well as T cells, are observed aggregated in the lesion area in rat model of EAE [96, 97]. The exact mechanisms of demyelination remained unclear. Glial cell proliferation during the process of MS may be the major effector in the development of a demyelinating plaque due to the composition of the inflammation infiltrating, as well as the dynamics of remyelination [98]. Early remyelinating lesions contained a mixture of infiltrated macrophages and microglia. Thin myelin sheaths were observed to surround the damaged axons [20, 34, 98], while in late remyelinating lesions only a few monomorphic populations of phagocytic macrophages presented in these lesions [98]. These studies suggested that insufficient myelin clearance in the CNS after MS onset may be involved in the failure of axonal regeneration. The relative contribution to disease progression of brain resident macrophages versus that of blood-borne macrophages remains controversial. Most studies favor the viewpoint that resident macrophages play a decisive role in disease progression and selective depletion of perivascular and meningeal macrophages using clodronate liposome injection in rat model of acute EAE slows disease progression [96]. Recent studies using bone marrow chimeras claimed that the number of microglia were several folds over blood-derived macrophages, though they shared equal phagocytic capacity [97]. These findings support the hypothesis that microglia are more efficient in phagocytosing myelin debris compared with peripheral macrophages. In an in vitro Wallerian degeneration model by cutting axons of the cortical explants, co-cultured microglia exposed to degenerated axon debris were first seen to engulf debris within15 h [99], whereas perivascular and meningeal macrophages infiltrating into the lesion area occurred at day 9 reached their maximum number at day 15 and decreased to normal level at day 24 post-EAE induction in rats [96]. The different types of macrophage activation in MS/EAE are closely correlated with the stage of the demyelinating activity and with the type of MS tissue [99]. In early-active MS lesion, massive activated macrophages aggregated at the plaque border and engaged in phagocytosing myelin degradation products; however, in late-active demyelination lesion, a small number of amoeboid-like macrophages were observed diffusely infiltrated in the lesion [98, 99]. The brain resident microglia are the main macrophages engaged in phagocytosis in the early stage of demyelination, while in the late stage a number of infiltrated blood-borne macrophages contribute to axon debris clearance. A variety of types of receptors are involved in MS, including Fc receptors, complement receptors, macrophage scavenger receptors, the galectin-3/MAC-2 receptors, α2-macroglobulin/low-density lipoprotein receptor, and mannose receptors [8]. In these receptor-linked pathways, TREM-2 signaling pathway has been the most well-studied. TREM-2 mediated phagocytosis of apoptotic neurons without release of pro-inflammation molecules in animal model of MS [35, 100]. Han et al. recently demonstrated that downregulation of CD47 promoted phagocytosis of myelin in a SIRP-α-dependent mechanism by using CD47^−/−^ EAE mice [101]. The capacity of macrophages/microglia to phagocytose degenerated myelin can be altered by environmental inflammatory mediators, such as IFN-γ, TNF-α, IL-4, IL-10, and so on [20]. This notion was confirmed by an in vitro study that investigated the effect of certain cytokines on phagocytosis of ^14^C-labeled myelin by cultured macrophages or microglia. In this study, TNF-α was shown to increase the phagocytic activity of microglia, but have no effect on macrophages [20]. TNF-γ was demonstrated to reduce removal of myelin by macrophage, on the contrary enhance debris clearance driven by microglia. IL-4 and IL-10 exerted a role of up-regulating phagocytosis in macrophages/microglia, while accompanied by a reduction of inflammatory response [20]. In demyelinated diseases, efficient removal of degenerated myelin is necessary to rebuild beneficial environment for remyelination and axon regeneration. Therefore, robust microglial phagocytosis might be critical in the recovery of MS/EAE.

### Microglial Phagocytosis in Brain Tumor

Nearly 18,000 patients die of primary tumors of the brain, and another 130,000 die from metastases neoplasm. Most primary brain tumors are of glial-cell origin, including glioblastoma multiforme, astrocytomas, oligodendrogliomas, ependymomas, and so on. Among which, malignant gliomas are the most common primary brain tumors and are highly aggressive with limited treatment efficiency. Macrophages/microglia were observed to present in tumors, tumor periphery, and contralateral tumor-free hemispheres, particularly around tumor margins [102, 103]. Badie and Schartner revealed that infiltrated microglia comprise 13.2 to 34.0 % of tumor mass, and the extent of microglia infiltration depends on the tumor type rather than its size [102]. Glioma-infiltrating microglia/macrophages show protumorigenic activity by upregulation of metalloprotease-II [104], but cannot secrete cytokines such as IL-1β, IL-6, and TNF-α, which is distinctive from the inflammatory phenotype [105]. In glioma microenvironment, normal human astrocytes (NHA), glioma cells, and microglia all show capability of phagocytosing glioma cells and specifically apoptotic tumor cells [106]. The phagocytosis index (the index to assess the phagocytic activity of phagocytes through counting of engulfed apoptotic cells) value of microglia showed about four-fold higher than that of NHA or glioma cells, indicating that microglia were the most efficient phagocytes in brain tumor [106]. IL-12 and LPS administration obviously enhance microglial phagocytotic activity through the TRAIL pathway [107]. In vitro study revealed that glioma cancer stem cells (gCSCs) dramatically inhibited phagocytosis by human microglia [108]. Further study regarding underlying mechanisms showed reversal of phagocytosis inhibition after STAT3 blockade by WP1066 and STAT3 siRNA in gCSCs, suggesting involvement of p-STAT3 pathway in inhibition of phagocytosis by gCSCs [108]. Microglia play a key role in phagocytosing tumor cells. This activity can be modulated by cancer cells or cancer environment. Whether microglial phagocytosis serves as cancer defense or cancer contribution still needs further investigation.

### Microglial Phagocytosis in Other Brain Diseases

Studies have shown that immune response is pivotal in prion disease [9, 109–112]. In prion disease, microglia become activated and take a decisive role in the progression of this disease [9]. Microglia in prion disease displayed low level of secretory profiles and elevated expression of phagocytotic machinery such as receptors for advanced glycation end products, TREM-2, and the scavenger receptors SAR2, CD68, and SRB(CD36) [109]. It is striking that intracerebral injection of LPS significantly stimulated synthesis and secretion of proinflammatory cytokines, but without alteration of PrPs clearance [109], which is thoroughly different from the generally accepted notion that microglial phagocytic state is positively in line with its activation degree [113]. Numerous findings demonstrated that microglia also participated in radiation induced neuronal damage [114–119]. Microglia with phagocytic morphology could be seen accumulated in the lesion cavity/spinal cord interface in irradiated spinal cord hemisection. Interestingly, microglial population rapidly decreased in the irradiated, lesioned hemisection when compared to nonirradiated animal [114, 118]. However, the exact mechanism underlying phagocytic process and the role of clearance by microglia in radiation induced brain injury needs further investigation.

## Conclusion

Microglia, as the resident macrophage in the CNS, play a critical role in the pathological and physiological processes of CNS diseases, sustaining the homeostasis of the local brain parenchyma. Even a minim disturbance can provoke the activation of microglia and following inflammation-induced neuronal damage. Most investigators favor that microglial phagocytosis exerts a beneficial effect in repair and regeneration. Variety types of receptors expressed on the cell surface of microglia are involved in mediating phagocytic process. These activated microglia exhibit an array of phenotypes and are governed by their surrounding environment. Activated homogenous macrophages infiltrate the blood–brain barrier and contribute to debris clearance in brain disease, such as ischemia, MS, trauma, and so forth. The relative contribution of resident microglia and peripheral macrophages remains controversial. The exact mechanism and functional aspect of phagocytosis by microglia needs further study, and modulation of microglial phagocytosis might be a potential therapeutic strategy for neurological diseases.
